# Cost analysis of limb salvage: comparing limb revascularisation and amputation in patients with Chronic Limb-Threatening Ischaemia (CLTI) at University Hospital Limerick

**DOI:** 10.1007/s11845-025-03885-9

**Published:** 2025-02-06

**Authors:** Anne Marie Toomey, Fiona Leahy, Helen Purtill, Norma O’Brien, Emer O’Donovan, Zeeshan Ahmed, Mekki Medani, Tony Moloney, Eamon G. Kavanagh

**Affiliations:** 1 School of Medicine, University of Limerick, Co. Limerick, V94 T9PX Limerick, Ireland; 2 Department of Vascular Surgery, University Hospital Limerick, St Nessan’s Rd, V94 F858 Dooradoyle Co. Limerick, Ireland; 3 Department of Mathematics and Statistics, University of Limerick, V94 T9PX Co. Limerick, Ireland; 4 Department of Blood Transfusion, University Hospital Limerick, St Nessan’s Rd, V94 F858 Dooradoyle, Co. Limerick, Ireland

**Keywords:** Amputation, Chronic limb threatening ischemia, Cost analysis, Length of stay, Revascularisation, Vascular procedures

## Abstract

**Background:**

The prevalence of peripheral arterial disease (PAD) is increasing globally. An increase in PAD in an ageing population inevitably results in an increase in incidence of Chronic Limb Threatening Ischemia (CLTI). Loss of a limb is a life-changing event with immeasurable cost to the individual, while the potential financial benefit of saving a limb is not well documented.

**Aims:**

The focus of this study was to estimate the cost associated with surgical interventions used in the treatment of CLTI compared with amputation.

**Methods:**

The cost to treat a CLTI diagnosis in 124 patients was analysed in an acute tertiary referral hospital over a 13-month study period. The analysis included staffing, medical devices used, number of blood components used and the length of stay. Statistical methods included descriptive statistical data and the Mann–Whitney U test.

**Results:**

The median cost, associated with length of stay, post-amputation and post-revascularisation (hybrid) was €61,313 [IQR = €44,417, €83,331] and €46,573 [IQR = €25,687, €58,554] respectively, *p* < 0.001.

The total median cost for length of stay for amputees in an acute hospital, rehabilitation and a prosthetic limb was €88,820 [IQR = €74,486, €110,248].

The median surgical cost of an amputation was €2,064 [IQR = €1,342, €2,866], whilst the median surgical cost of a revascularisation procedure (hybrid) was €5,966 [IQR = €4,380, €7,723], *p* < 0.001, inclusive of total blood components transfused.

**Conclusion:**

Revascularisation surgical interventions are more expensive than amputation, however, the length of stay, rehabilitation and prosthetic limb costs, for a patient undergoing a major limb amputation, is significantly more costly.

## Background

There is an increasing prevalence, world-wide, of peripheral arterial disease (PAD) which is attributable to an ageing population and the increasing prevalence of Diabetes Mellitus [1]. An increase in PAD prevalence will therefore increase the incidence of Chronic Limb Threatening Ischemia (CLTI) [2]. This study aims to investigate the cost implications of treating patients with CLTI.

CLTI is defined as the existence of peripheral arterial disease with evidence of pain, gangrene or lower limb ulceration lasting for a duration of more than 2 weeks [1]. It is a clinical diagnosis which may be supplemented by hemodynamic readings i.e., ankle pressure of < 50 mmHg, toe pressure < 30 mmHg, an ankle brachial index (ABI) < 0.4 and transcutaneous oximetry where appropriate. Staging of CLTI, as per the European Society of Vascular Surgery (ESVS), is accomplished via the Wound, Ischaemia and foot Infection (WIfI) classification system. [1].

Surgical interventions for patients with CLTI include primary amputation, endovascular revascularisation, open revascularisation and hybrid revascularisation i.e., combined endovascular and open techniques. Interventions are chosen based on fitness and risk to the patient, extent of the disease and the anatomical pattern of disease [1].

Decisions for intervention are guided by the WIfI classification [1]. In addition, the Global Limb Anatomic Staging System (GLASS) classification and the PLAN (Patient risk, Limb status, ANatomical Pattern) framework are used to complete the pathway for treatment of CLTI [1]. Moreover, bedridden patients, those with advanced dementia and other conditions that may preclude successful rehabilitation, where ambulation post-operation is not expected, may be offered amputation as the definitive treatment [3, 4].

Clinical outcomes vary greatly for each intervention. The 5-year mortality rate for major amputations i.e., below knee (BKA) and above knee (AKA), is reported to range from 30 to 70%, with AKA having the lowest survival [5, 6]. A patient with a major limb amputation and concomitant Type II diabetes is noted to have a much lower survival rate than some malignant diseases [6].

With respect to bypass revascularisation, the preferred conduit is an autogenous vein graft for above and below the knee interventions [1, 7]. If autogenous grafts are not available due to previous harvesting/stripping or unsuitable for use due to the presence of varicose veins or thrombophlebitis, prosthetic grafts may be used; these are more suitable for above the knee interventions as the patency of below knee prosthetic grafts is extremely limited. [1, 8].

Furthermore, the BASIL-1 trial highlighted the complexity of bypass interventions, it was observed that prosthetic grafts had a worse amputation free survival (AFS) and overall survival when compared to plain balloon angioplasty (PBA) alone. Whilst those who had a PBA in advance of a prosthetic bypass intervention had an even more negative AFS outcome. [8].

Finally, endovascular revascularisation procedures have their own limitations and selectivity, for example stenting in the femoral-popliteal vessels is not routinely performed, and balloon angioplasty is the preferred option for infra-popliteal vessel occlusions [1]. Additional endovascular options have increased in recent years and now include mechanical atherectomy, lithotripsy, intravascular ultrasound as well as drug-eluting balloons and drug-eluting stents, all of which have significant costs. [9].

With several options available to vascular surgeons for treatment of CLTI, there is a variation in cost for each procedure and the subsequent length of stay in acute hospitals. This study aims to investigate the cost implication of each of these procedures and the length of stay.

## Materials and methods

This study was a retrospective observational study which focused on *n* = 124 patients; these patients satisfied the following criteria:

- Confirmed CLTI diagnoses;
- Infra-inguinal disease;
- Admitted to University Hospital Limerick (UHL) for surgical treatment of CLTI between March 1st 2022 and March 31st 2023.

Surgical procedures included in this study were as follows:

- Revascularisation procedures
  - Angioplasty/stenting
  - Lower limb bypass surgery
  - Femoral endarterectomy
  - Embolectomy (lower limb)
- Major limb amputation
  - Below knee amputation (BKA)
  - Above knee amputation (AKA)
  - Through knee amputation (TKA)

Any re-interventions/surgical revisions post March 31st 2023, for these *n* = 124 patients, were included in the length of stay and cost calculations.

### Study setting

The study took place in University Hospital Limerick (UHL) which is a model 4 tertiary referral centre with 533 inpatient beds [10]. UHL is located in the Mid-West Health region which encompasses a population of 523,700 (value projected from 2016 data and includes all age groups) [11]. With regards to the UHL Vascular Department, the department comprises 3.5 whole time equivalent (WTE) vascular surgeons.

Patients may have presented to the UHL vascular department with non-healing ulcers, gangrene, claudication, or lower limb rest pain. These patients were, subsequently, treated conservatively or surgically for CLTI.

Decision to proceed with a particular type of surgical intervention is typically aided by the PLAN framework. The PLAN framework describes the optimum pathway for the treatment of patients that have potential for revascularisation. [1].

Amputation of lower limbs i.e., AKA, BKA or TKA is chosen as a last resort or if the patient is deemed bedbound.

### Data collection

A chart review was performed to collate population demographics of the *n* = 124 patients. Information relating to smoking status and comorbidities i.e., Diabetes Mellitus (DM), Ischemic Heart Disease (IHD), Renal Disease, Cerebral Vascular Accident (CVA), Transient Ischemic Attack (TIA), Neurological and Cognitive, were extracted as part of this review.

Length of stay, post-operatively, was obtained from the integrated Patient Management System (iPMS). Length of stay for every procedure performed on the *n* = 124 patients, from March 1st, 2022, was collected; this included any re-interventions performed on this patient cohort.

Medical devices implanted i.e., stents and synthetic grafts, and balloon catheter devices employed during revascularisation procedures were obtained and collated from the Theatre Implant Order Book.

Finally, the number of blood components administered to the group of interest, pre- and post-operatively, were extracted from a register provided by the Blood Transfusion Department in UHL.

### Cost analysis

#### Cost calculation for medical devices and procedures

Costing for medical devices implanted and balloon catheters were supplied by representatives of the individual medical device companies supplying these devices.

Costing for processing and the sterilisation of amputation and percutaneous angiogram kits were supplied by the Theatre Clinical Nursing Managers (CNMs). Processing of this equipment was estimated to be €200 for amputation kits and €40 for percutaneous angiogram kits.

Staffing of the operating theatre, for each procedure, was calculated using the average HSE salaries across all grades. One consultant surgeon and one anaesthetist consultant fee were averaged to be €130/Hr each, one SpR/Registrar was averaged at €38/Hr, one Senior House Officer was €31/Hr, one Clinical Nurse Manager (CNM) and three nursing staff was averaged at €97/Hr [12]. In the event where an embolisation procedure was performed, an additional consultant fee of €130/Hr for an interventional radiologist was included.

Duration for each procedure was extracted from the theatre time-in and time-out log-book. If a procedure duration was not recorded, a duration for a similar procedure was incorporated. A missing duration occurred for 3% of cases. Of note, the average duration for each procedure was as follows:

- Amputation = 3.1 h;
- Hybrid = 5.9 h;
- Open procedures = 5.4 h;
- Endovascular = 3.4 h.

Blood component costs were provided by the Blood Transfusion Department in UHL; this cost is detailed Appendix 1 in Table 4.

Prosthetic limb and shrinker costs were obtained from invoice data documented in the manufacturer’s sales quotation.

#### Cost calculation for length of stay

The cost associated with length of stay was calculated using data from the Diagnosis Related Group (DRG) for 2022 and 2023 [13, 14]. Costing for rehabilitation was obtained from the 2020 DRG [15]; data for rehabilitation was not calculated in 2022 or 2023 DRG documentation.

Furthermore, the ICU length of stay was collated and included in the overall costs; this is presented in the \* MERGEFORMAT Cost Calculation for Length of Stay Section. The ICU associated cost calculation was based on the DRG major complexity category, which approximately doubled the cost of length of stay. Note, not all patients required an admission to ICU.

The DRG quantifies hospital activity into an average cost per case/diagnoses; the DRG is derived from a Casemix which comprises the monitoring of activity and costs between hospitals [16]. DRG categories used in this study are documented in Appendix 1 Table 5.

DRG data is presented in terms of base price and weighted units or relative values. Base prices for 2022 and 2023 were €5,496 and €6,101, respectively whilst the 2020 base price was €5,107. Relative values represent the weighted value for a diagnosis; these are then used in conjunction with the base price to obtain the cost of a case per day. The DRG calculates an inlier patient price, this is between a low and high boundary, i.e., 2 and 9 days. For a length of stay more than 9 days it is considered a high outlier duration and is accounted for in the relevant calculations. Relative values and an example calculation are presented in Appendix  1 Tables 6 and 7.

Note, DRG relative values remain the same between years 2022 and 2023, however the base price varies i.e., €5,496 for year 2022 and €6,101 for year 2023.

Lastly, for a patient undergoing a hybrid procedure, for example a bypass and a distal angioplasty, the summative cost of these procedures is extracted and used in the cost estimates.

### Statistical analysis

Categorical data are summarised as number (percentage). Quantitative data were assessed for skewness through visual inspection of histograms and Q-Q plots.

In this study, length of stay, the associated cost for length of stay, cost of procedures and blood component costs have a skewed distribution and are therefore summarised as median and inter quartile range (IQR). Differences in these variables between the amputation group and each of the three revascularisation groups (endovascular, open and hybrid procedures) were examined using the non-parametric Mann–Whitney U test, where tests were two-tailed and a *p*-value < 0.05 deemed statistically significant [17].

Software used to perform the statistical calculations was SPSS version 28 [18].

## Results

This section details the following:

- Population demographic;
- Length of stay in UHL;
- Number of blood components transfused;
- Cost associated with length of stay;
- Procedural cost including cost of blood components;
- Rehabilitation and cost of prosthesis, if applicable.

**Fig. 1:**
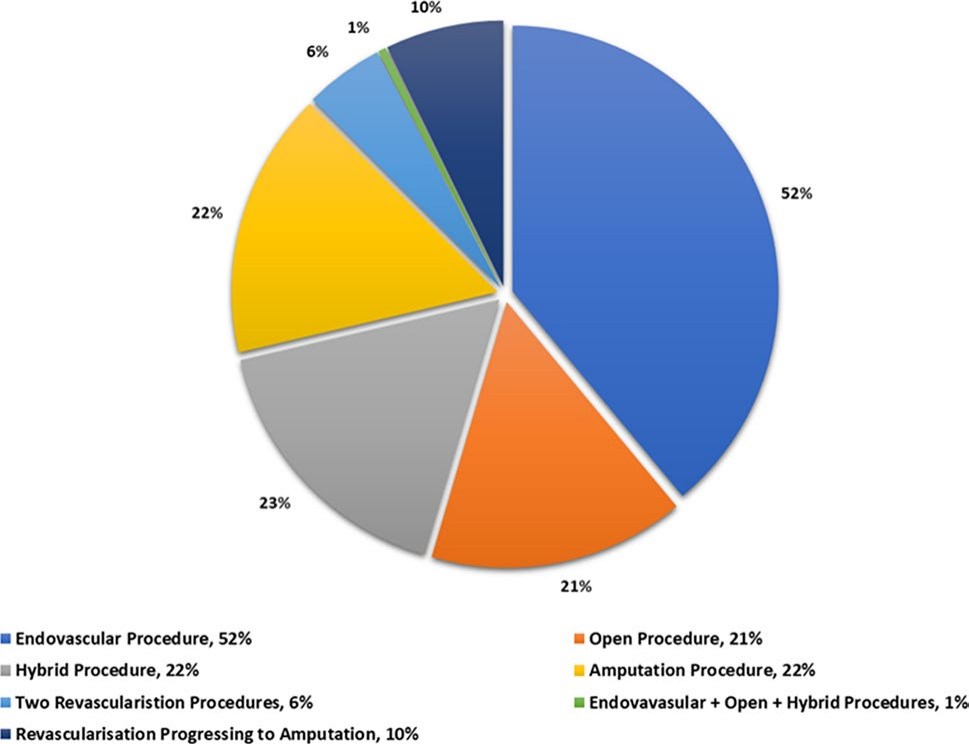
Percentage of patients in each cohort

The total number of patients included in the study was *n* = 124 patients. Patients undergoing revascularisation was *n* = 109 patients and those undergoing amputation was *n* = 27 patients. The number of patients who had a revascularisation procedure, within the study period, and progressed to having an amputation was *n* = 12 patients. A percentage breakdown of patients undergoing amputation and revascularisation procedures i.e., endovascular, open and hybrid, is presented in Fig. 1.

### Demographic

Baseline demographics for this study’s CLTI cohort are presented in Table 1.

**Table 1:** Baseline demographics for patients undergoing revascularisation and major limb amputations

Category	Successful revascularisation *n* = 109	Major limb amputation *n* = 27
Male	78 (72%)	23 (85%)
Age (> 65 years old)	92 (84%)	19 (70%)
Smoker/Ex Smoker	43 (39%)	16 (59%)
Diabetes Mellitus	43 (39%)	12 (44%)
IHD	34 (31%)	11 (41%)
CVA	3 (3%)	8 (30%)
TIA	5 (5%)	2 (7%)
Renal Disease	17 (16%)	6 (22%)
Neurological	7 (6%)	10 (37%)
Cognitive	2 (2%)	7 (26%)
Mortality	11 (10%)	4 (15%)
Previous Revascularisation Procedure(s) ^(b)^	37 (34%)	22 (81%)

a. Results are presented in the following format: Number of patients (percent of that cohort)

b. Number of patients who had more than one revascularisation procedure or who had revascularisation performed pre-amputation

The population of interest comprises largely over 65-year-old males. Over half of the amputees, in this study, are noted to be smokers or ex-smokers.

Additionally, 81% of amputees had previous revascularisation procedures performed, within and beyond the study period.

### Post-operative status of patients in the amputee cohort

56% of patients who underwent an amputation received or are awaiting a prosthesis. 63% of this cohort were successfully discharged to rehabilitation units ﻿(Table 2). Note, a rehabilitation unit refers to a Model 2 or Model 3 Hospital with rehabilitation support or the National Rehabilitation Hospital (NRH).

**Table 2:** Prosthesis and rehabilitation status

Category	Limb Amputation, *n* = 27
Received/Awaiting a prosthesis	15 (56%)
Attended rehabilitation	17 (63%)

### Length of stay

On assessment of the cumulative length of stay for this cohort, it is evident that length of stay (days), post-intervention, is significantly higher for those who have undergone an amputation. Comparing the amputation group against each of the revascularisation groups, the median length of stay post-amputation is 29 days [IQR = 13, 39] (median [interquartile range (IQR)]), whilst the median length of stay post-revascularisation procedures are as follows:

- Endovascular Procedures: 5 days [IQR = 2, 15], *p* = <0. 001;
- Open Procedures: 7.5 days [IQR = 6, 20], *p* = 0.002;
- Hybrid Procedures: 10.5 days [IQR = 5.5, 27.5], *p* = 0. 007.

**Fig. 2:**
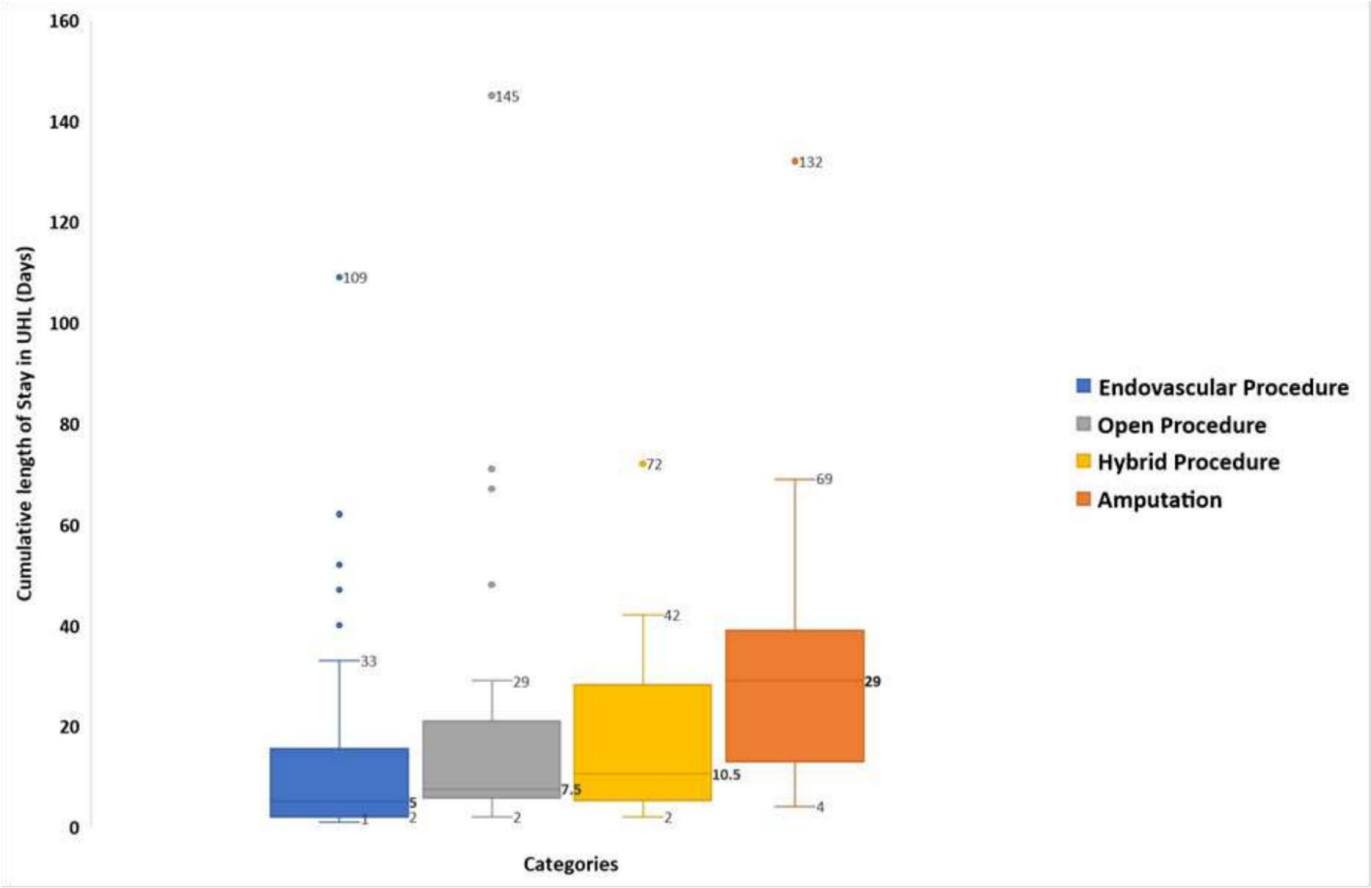
Medians and IQR for cumulative length of stay for revascularisation versus amputation

Medians and interquartile ranges for each group are presented in Fig. 2.

**Fig. 3:**
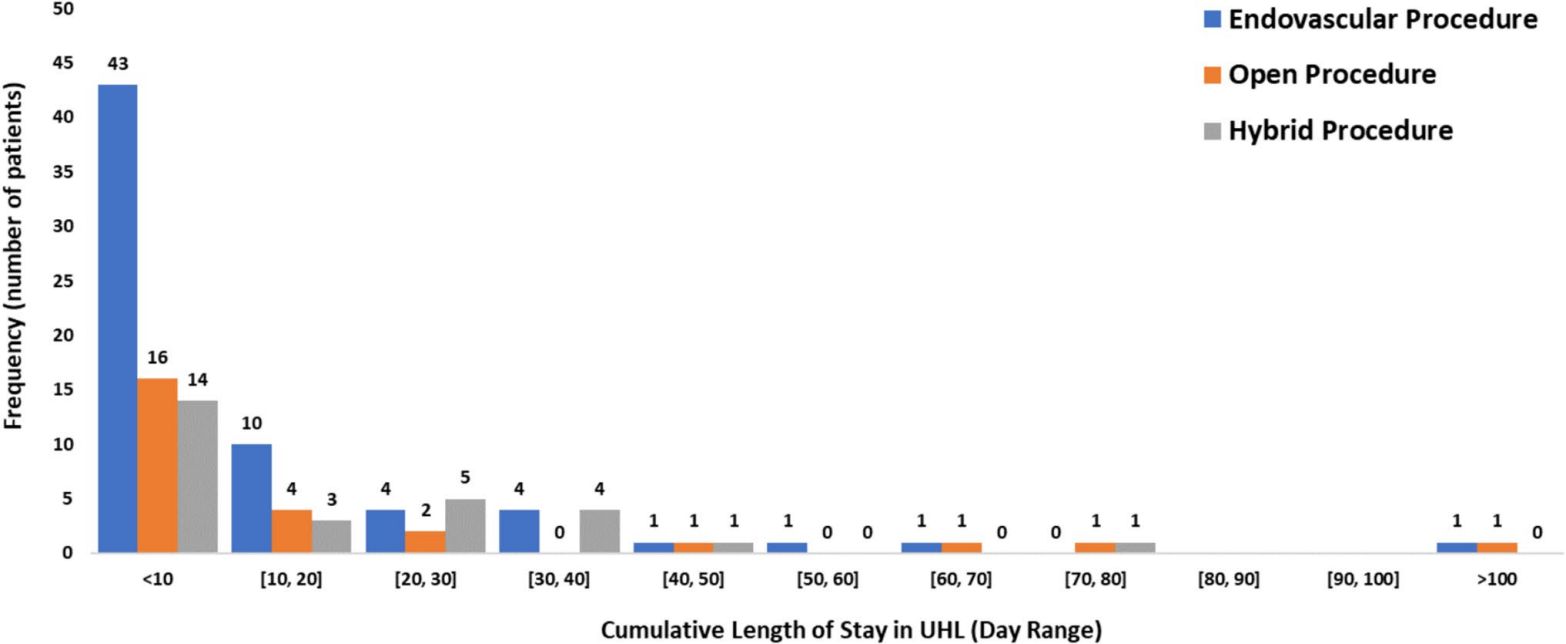
Revascularisation groups—cumulative length of stay

**Fig. 4:**
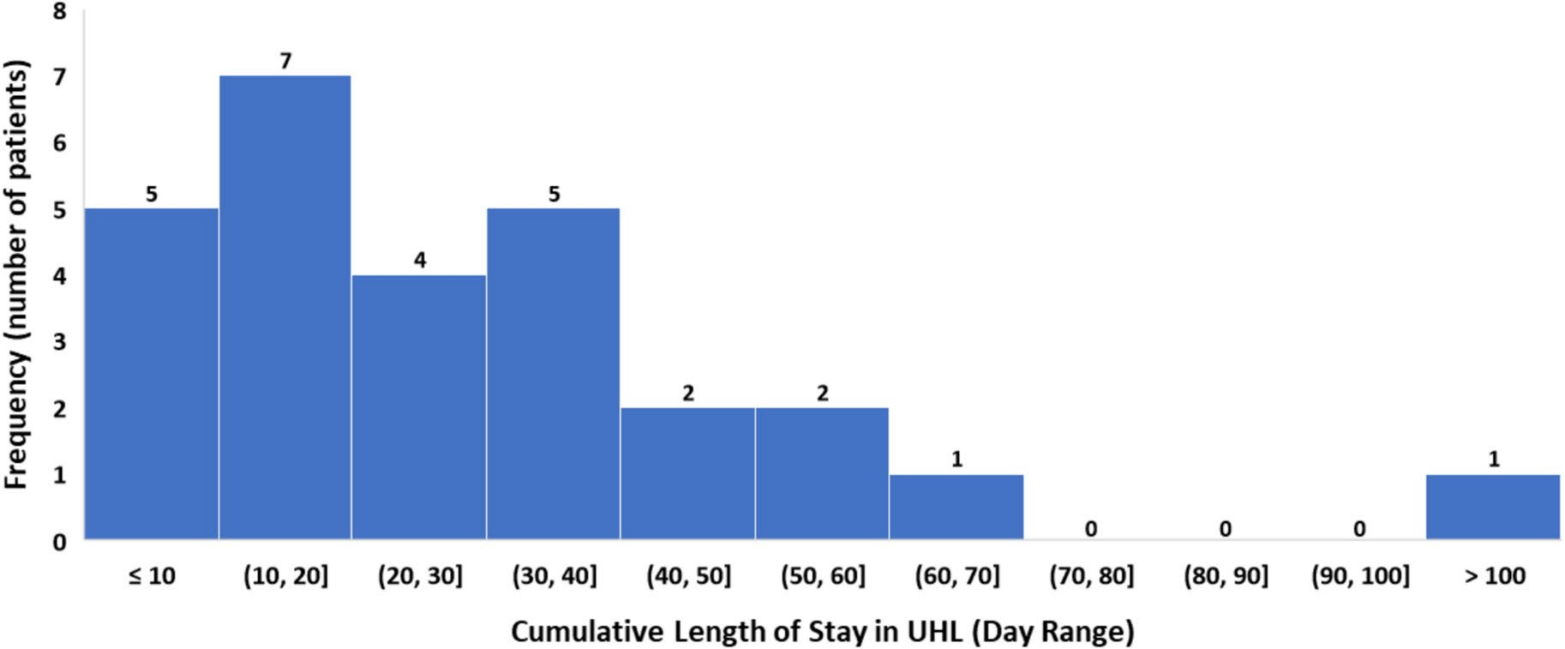
Amputee group—cumulative length of stay in UHL

The cumulative length of stay post-intervention and the frequency of patients staying in hospital for both revascularisation procedures and amputations are presented in Figs. 3 and 4, respectively. Once more, it is observed that patients undergoing a major limb amputation have a significantly longer length of stay in comparison to patients undergoing revascularisation.

**Fig. 5:**
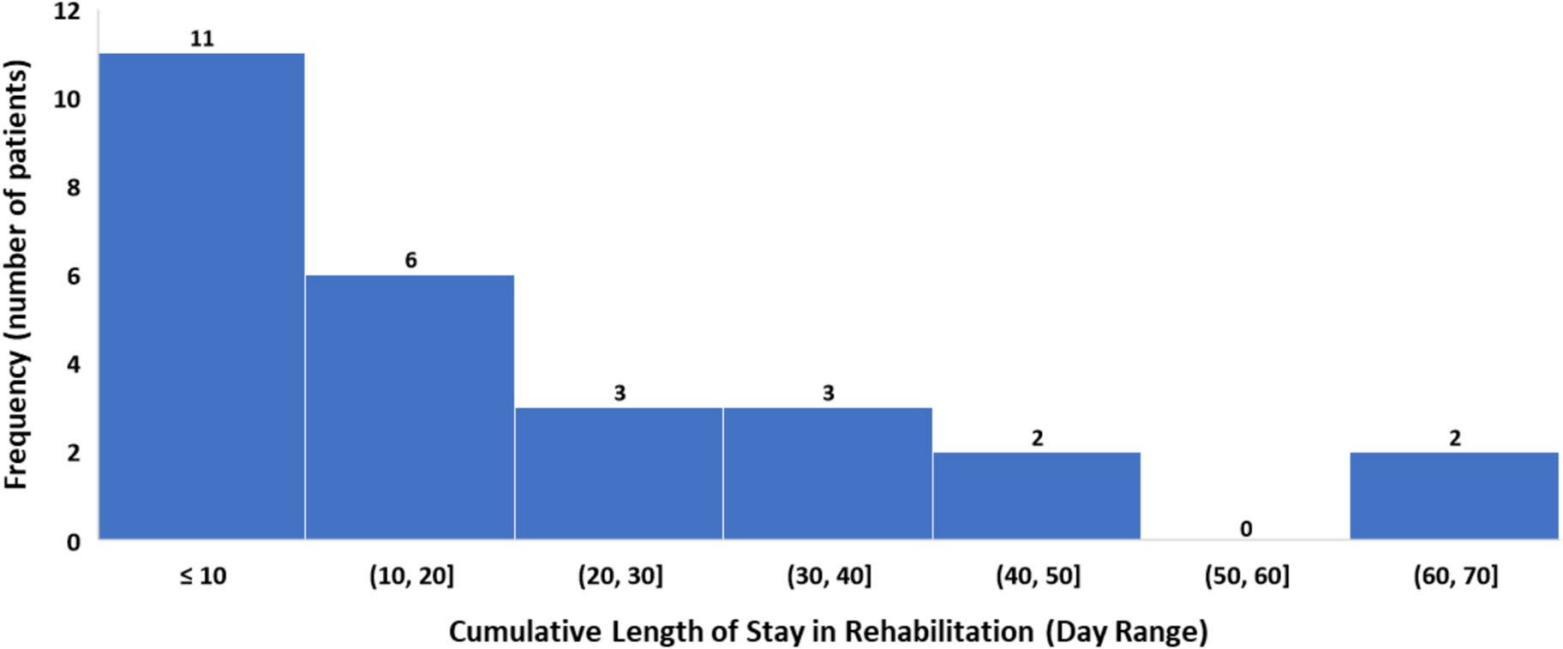
Amputee group – cumulative length of stay in rehabilitation

The cumulative length of stay and the frequency of patients admitted for rehabilitation is presented in Fig. 5.

### Blood components

Blood components, which includes Red Cells, Platelets, Fibrinogen, SD Plasma or Albumin, were administered to *n* = 42 individual patients (34%) across all groups i.e., amputation and revascularisation groups..

Moreover, 52% (*n* = 14) of amputees received blood transfusions whilst those undergoing revascularisation received the following:

- Endovascular Procedures: 29% (*n* = 19);
- Open Procedures: 35% (*n* = 9);
- Hybrid Procedures: 39% (*n* = 11).

**Fig. 6:**
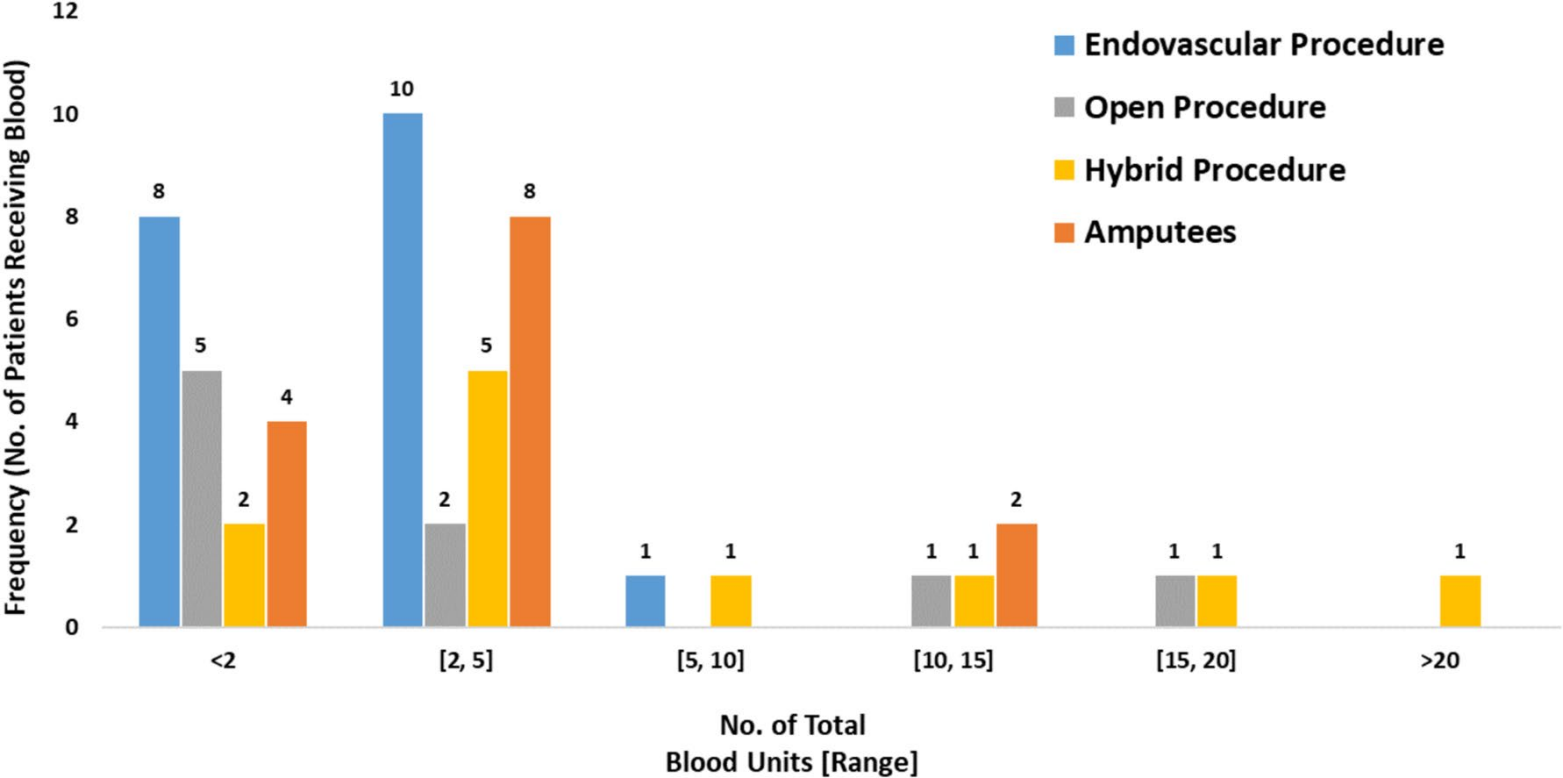
Frequency of patients receiving blood

The quantity of patients receiving blood transfusions is depicted in Fig. 6.

The number of blood components, administered pre, intra- and post-surgical intervention, were collated and analysed. It was observed that there was no statistically significant difference between amputation and revascularisation procedures.

Comparing the amputation group against each of the revascularisation groups, the median number of blood components administered pre- and post-amputation were 2 components [IQR = 1, 4], whilst the number of blood components administered pre- and post-revascularisation procedures were as follows:

- Endovascular Procedures: 2 components [IQR = 1, 3], *p* = 0.365;
- Open Procedures: 1 component [IQR = 1, 4], *p* = 0.579;
- Hybrid Procedures: 3 components [IQR = 2, 13], *p* = 0.303.

Blood components administered and median values across all groups are provided in Table 3.

**Table 3:** Blood components administered to patients undergoing revascularisation and lower limb amputations

Group	Endovascular procedures *n* = 65	Open procedures *n* = 26	Hybrid procedures *n* = 28	Lower limb amputees *n* = 27
No. of Patients receiving Blood Transfusions ^(a)^	19 (29%)	9 (35%)	11 (39%)	14 (52%)
No. of Blood Components Administered	42	28	44	54
Median and IQR	2 [1, 3]	1 [1, 4]	3 [2, 10]	2 [1, 4]

a. Results are presented in the following format: Number of patients (percent of that cohort)

### Cost Analysis

#### Cost of surgical interventions

The cumulative cost of surgical interventions was analysed for both revascularisation and amputation procedures; the cost accounts for the initial surgical intervention and any surgical revisions after March 1st, 2022 and for blood components administered during the patient’s length of stay.

The cost of surgical interventions for revascularisation and major limb amputation demonstrates a different trend to that of length of stay. Comparing the amputation group against each of the revascularisation groups, the median surgical intervention cost for amputation, including the cost of blood components administered, was €2,064 [IQR = €1,342, €2,866], whilst the medians for cost of revascularisation interventions, for the population of interest were as follows:

- Endovascular Procedures: €3,384 [IQR = €2,314, €5,340 ], *p* = <0.001;
- Open Procedures: €3,631 [IQR = €2,364, €5,121], *p* = <0.001;
- Hybrid Procedures: €5,966 [IQR = €4,380, €7,723 ], *p* = <0.001.

The medians and *p*-values indicate that there is a statistically significant difference in the cost of amputation and each of the revascularisation interventions.

**Fig. 7:**
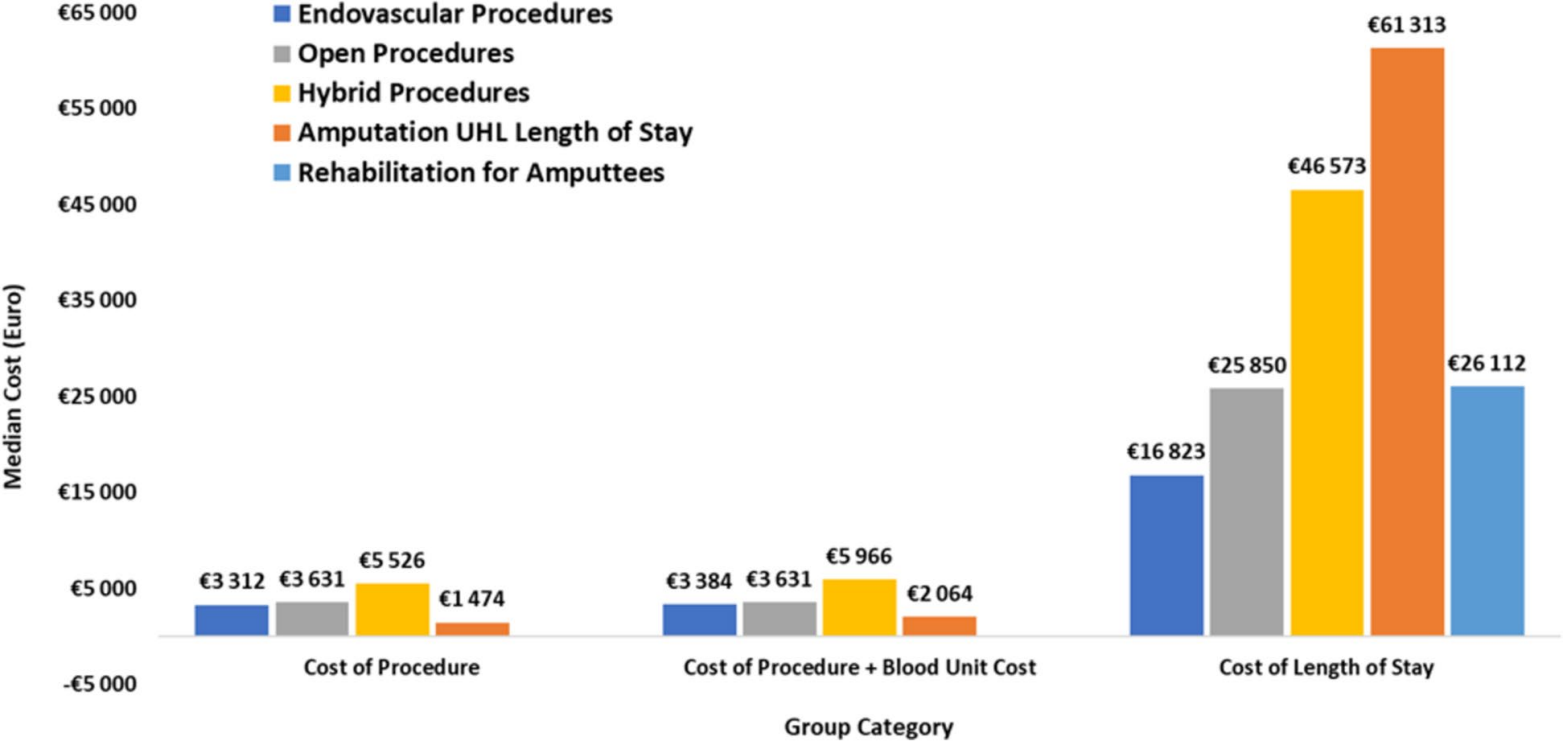
Comparison of medians for cost of procedure, cost of procedure + blood component cost and cost of length of stay for UHL and rehabilitation

Medians for surgical intervention costs are depicted in Fig. 7 for revascularisation and amputation procedures with and without the inclusion of blood components administered.

#### Cost of length of stay

The cost associated with length of stay was calculated and analysed for both revascularisation and amputation cases; the cumulative cost of length of stay includes post-intervention length of stay cost and length of stay cost associated with any re-interventions.

Comparing the amputation group against each of the revascularisation groups, there is a statistically significant difference in amputation and revascularisation length of stay cost. The median length of stay cost post-amputation in UHL was €61,313 [IQR = €44,417, €83,331], the medians for length of stay cost post-revascularisation were as follows:

- Post-Endovascular Procedures: €16,823 [IQR = €10,998, €33,889], *p* = <0.001;
- Post-Open Procedures: € 25,850 [IQR = €23,287, €58,048], *p* = <0.001;
- Post-Hybrid Procedures: €46,573 [IQR = €25,687, €58,554], *p* = <0.001.

Additionally, the cost of rehabilitation for amputees was calculated, the median cost of length of stay in a rehab facility was €26,112 [IQR = €22,966, €31,954].

Furthermore, the total median cost for length of stay for amputees in an acute hospital, rehabilitation and a prosthetic limb was €88,820 [IQR = €74,486, € 110,248].

Medians for length of stay cost for post-revascularisation and post-amputation are depicted in Fig. 7.

## Discussion

This is the first assessment of cost and length of stay following surgery for CLTI in an Irish setting. While the cost of revascularisation procedures is rising rapidly due to evolving endovascular technologies, post-amputation rehabilitation remains constrained by limited dedicated specialist rehabilitation beds and a significant waiting time for services provided by the National Rehabilitation Hospital. It was deemed a matter of interest to assess and compare the cost of performing revascularisation procedures and amputation procedures, as well as length of stay after each intervention.

As expected, the length of stay was significantly shorter for patients undergoing revascularisation procedures; in fact, the length of stay for an amputee post-intervention was 2.8 times greater than that of the length of stay post-hybrid revascularisation procedures. Moreover, the cost associated with the length of stay for amputees was 1.3 times greater than the cost of length of stay post-hybrid revascularisation procedures in the acute hospital.

In contrast, the trend reverses for the cost of surgical intervention, an amputation procedure costs 2.9 times less than a hybrid revascularisation procedure. The increased cost for revascularisation is attributed to longer operating times for these procedures i.e., bypass or endarterectomy and the cost of medical devices implanted or used i.e., stents, synthetic grafts, and balloon catheters.

On assessment of the number of blood components administered, it was found that there was no statistically significant difference between the revascularisation groups and the amputation group.

In the management of a patient’s CLTI diagnosis, it is important that we are aware of all the inputs required to treat a patient i.e., social aspects, medical and surgical interventions and the cost involved at every level. This awareness aids the healthcare professional to comprehend and quantify, completely, the resources required to effectively treat their patient.

### Limitations

This study includes several limitations, most importantly is that of allied healthcare contributions i.e., physiotherapy, occupational therapy, and podiatry. Allied healthcare allows for early presentation of patients and earlier recovery time. At UHL there is no dedicated service to vascular patients and therefore these contributions could not be assessed accurately.

Lastly, information on wound healing post-operation has also not been included in this study. Incomplete wound healing or development of a wound infection may result in future surgical interventions i.e., debridement or amputation revisions and thus could increase the cost of treatment.

## Conclusion

In our study, set in the UHL Vascular Department, we concluded that whilst revascularisation surgical interventions are more expensive when compared to amputation procedures, the cost associated with the length of stay of a patient undergoing a major limb amputation is significantly more expensive.

It is well described in published studies, that revascularisation is the more cost-effective approach in the management of patients who have symptomatic CLTI. It remains important, however, to assess/audit regularly the cost involved in such procedures, so that healthcare professionals can comprehend the full context of the management of these patients [19, 20]. While it would be exceedingly difficult to estimate the cost of limb loss to the individual and to society in general, it would seem a timely reminder that the cost of bed days alone makes efforts to save limbs through revascularisation very worthwhile, dwarfing the costs of medical devices.

## Data Availability

Raw data, for this assessment, is not publicly available to preserve individuals’ privacy under the European General Data Protection Regulation.

## Appendix 1

### Cost Analysis

See Tables 4, 5, 6, and 7 here.

**Table 4:** Blood component cost

Blood unit cost blood unit cost blood component type	Blood component cost
Red cells	€295.00
Albumin	€54.60
Platelets	€700.00
Fibrinogen	€440.00
SD plasma	€116.00

**Table 5:** DRG code description [21]

DRG code	Description	Assigned procedure
F14A	Vascular Interventions, Except Major Reconstruction, W/O CPB Pump, Major Comp	Bypass and Endarterectomy
F14B	Vascular Interventions, Except Major Reconstruction, W/O CPB Pump, Interm Comp	Angioplasty
F14C	Vascular Interventions, Except Major Reconstruction, W/O CPB Pump, Minor Comp	Angiogram
F11A	Amputation, Except Upper Limb and Toe, for Circulatory Disorders, Major Comp	Not used in calculation
F11B	Amputation, Except Upper Limb and Toe, for Circulatory Disorders, Minor Comp	Limb Amputation
F13A	Amputation, Upper Limb, and Toe, for Circulatory Disorders, Major Complexity	Not used in calculation
F13B	Amputation, Upper Limb, and Toe, for Circulatory Disorders, Minor Complexity	Toe Amputation
Z60A [ \* MERGEFORMAT 17]	Rehabilitation, Major Complexity	Rehabilitation for Amputees

**Table 6:** DRG cost [14] – relevant values (RV) and factors from the DRG 2023^(b)^

DRG code	Inpatient inlier price ^(a)^	Same day RV	One day RV	Multi low RV	Inlier RV	Multi high RV	Average length of stay (Days)
F14A	€25,848	0.937	4.237		4.237	0.174	18.4
F14B	€12,210	0.706	2.001		2.001	0.156	8.2
F14C	€7,739	0.456	1.268		1.268	0.179	4.3
F11B	€41,123	1.363	1.494	0.262	6.740	0.184	37.6
F13B	€11,796	0.623	1.060		1.934	0.155	10.4

a. Inpatient Inlier Price equates to diagnoses price for a length of stay > 2 days and < 9 days.

b. 2023 DRG RV values are equal to 2022 DRG RV values, however, base price varies between 2022 and 2023.

**Table 7:** Calculation – DRG calculation for length of stay [14]

Length of stay	Calculation	Example: DRG Code F14A – Major Vascular Intervention
One day	$Base Price x One Day RV$	$\in6,101x4.237=\in25,848$
Between 2 and 9 days	$Base Price x Inlier RV=Inpatient Inlier Price$	$\in6,101x4.237=\in25,848$
20 days	$Base Price x (Inlier RV+\left(LoS-9\right) x Multi High RV$	$4.237+\left(20-9\right)\times\in6,101=\in37,527$

## References

[1] Conte M, Bradbury A, Kolh P (2019) ‘Global vascular guidelines on the management of chronic limb ischemia’, clinical practice guideline document. European Journal of Vascular Endovascular Surgery 58(1):S1–S109>E33. 10.1016/j.ejvs.2019.05.006 31182334 10.1016/j.ejvs.2019.05.006 PMC8369495

[2] Hart, O., Xue, N., Davis-Havill, B., et al. (2022) The incidence of chronic limb threatening ischemia in the midland region of New Zealand over a 12-year period. Journal of Clinical Medicine 11(12):3303. 10.3390/jcm11123303 10.3390/jcm11123303 PMC9225294 35743374

[3] Aziz, H., Branco, B.C., Braun, J.et al. (2015) The influence of do not resuscitate status on the outcomes of patients undergoing emergency vascular operations. J of Vascular Sur 61(6):1538–1542. 10.1016/j.jvs.2014.11.087 10.1016/j.jvs.2014.11.087 25704406

[4] Siracuse JJ, Jones DW, Meltzer EC et al (2015) ‘Impact of “do not resuscitate” status on the outcome of major vascular surgical procedures. Annals of Vascular Sur 29(7):1339–1345. 10.1016/j.avsg.2015.05.014 10.1016/j.avsg.2015.05.014 26169461

[5] Subramaniam B, Pomposelli F, Talmor D, Park KW (2005) ‘Perioperative and long-term morbidity and mortality after above-knee and below-knee amputations in diabetics and nondiabetics. Anesth Analg 100(5):1241–1247. 10.1213/01.ANE.0000147705.94738.31 15845661 10.1213/01.ANE.0000147705.94738.31

[6] Fortington, L.V., Geertzen, J.H., van Netten, J.J. et al. (2013) Short and long term mortality rates after a lower limb amputation. Euro J Vascular Endovascular Sur 46(1):124–131. 10.1016/j.ejvs.2013.03.024 10.1016/j.ejvs.2013.03.024 23628328

[7] Menard, M.T., Farber, A., Assmann, S.F. et al (2016) ‘Design and rationale of the best endovascular versus best surgical therapy for patients with critical limb ischemia (BEST‐CLI) Trial. J Am Heart Asso 5(7). 10.1161/jaha.116.003219. 10.1161/JAHA.116.003219 PMC5015366 27402237

[8] Bradbury A.W., Adam D.J., Bell J. et al (2010) Bypass versus angioplasty in severe ischaemia of the leg (BASIL) trial: Analysis of amputation free and overall survival by treatment received. J Vascular Sur, 51(5). 10.1016/j.jvs.2010.01.074 10.1016/j.jvs.2010.01.074 20435259

[9] Beckman JA, Schneider PA, Conte MS (2021) Advances in revascularization for peripheral artery disease: Revascularization in PAD. Circu Res 128(12):1885–1912. 10.1161/circresaha.121.318261 10.1161/CIRCRESAHA.121.318261 34110904

[10] HSE (n.d.), ‘UL Hospitals Group: Facts and Figures’ available: https://healthservice.hse.ie/healthcare-delivery/ul-hospitals-group/about-ul-hospitals-group/ul-hospital-group-facts-and-figures.html [accessed: 12 Aug 2023]

[11] Department of Health, The HRB national drugs library - drugs and alcohol (2022), ‘Health in Ireland, Key Trends in 2022’ available at: https://www.drugsandalcohol.ie/37620/1/Health%20in%20Ireland%20Key%20Trends%202022.pdf [accessed: 12 Aug 2023]

[12] HSE (2022), ‘Health Sector - Consolidated Salary Scales in Accordance with The Fempi Acts, The Public Service Agreements and The Public Service Pay and Pensions Act 2017’, available: https://assets.hse.ie/media/documents/Pay_scales_for_2.2.22_and_1.10.22_adjustments_V2.pdf [accessed: 29 Jul 2023]

[13] Health Pricing Office (2022), ‘ABF 2022 admitted patient price list DRG prices for inpatients and day cases 2022’, available: https://www.hpo.ie/abf/ABF2022AdmitedPatientPriceList.pdf [accessed: 29 Jul 2023]

[14] Health Pricing Office (2023), ‘ABF 2023 admitted patient price list DRG prices for inpatients and day cases 2023’, available: http://www.hpo.ie/abf/ABF2023AdmittedPatientPriceListv1.pdf [accessed: 29 Jul 2023]

[15] Health Pricing Office (2020), ‘ABF 2020 Admitted Patient Price List DRG Prices for Inpatients and Daycases 2020’, available at: https://www.hpo.ie/abf/ABF2020AdmittedPatientPriceList.pdf [accessed: 31 Oct. 2021].

[16] Department of Health and Children (2023), ‘Casemix measurement in Irish Hospitals? a broad outline of the main features / Casemix Unit, available: https://www.lenus.ie/bitstream/handle/10147/46324/1179.pdf;jsessionid=BBE8022AE8ACC698960ADD53450622A3?sequence=1 [accessed: 30 Jul 2023]

[17] Tenny, S. and Abdelgawad, I. (2022) ’Statistical significance’ [online] Nih.gov. available at: https://www.ncbi.nlm.nih.gov/books/NBK459346/ [accessed: 21 Jan 2024]

[18] IBM Corp (2021) IBM SPSS Statistics for Windows, Version 28.0. Armonk, NY: IBM Corp

[19] Tang L et al (2018) Cost analysis of initial treatment with endovascular revascularization, open surgery, or primary major amputation in patients with peripheral artery disease. J Endo Therapy: An Afficial J Inter Soc Endo Spec 25(4):504–511. 10.1177/1526602818774786 10.1177/1526602818774786 29756521

[20] Shan LL et al (2022) A systematic review of cost-utility analyses in chronic limb-threatening ischemia. Anns Vascular Sur 85:9–21. 10.1016/j.avsg.2022.04.036 10.1016/j.avsg.2022.04.036 35561892

[21] Independent Hospital Pricing Authority (2019) ‘Australian Refined Diagnosis Related Groups Version 10.0 Final Report’ available: https://www.ihacpa.gov.au/sites/default/files/2022-08/AR-DRG%20Version%2010.0%20Final%20Report_0.pdf [accessed: 29 Jul 2023]

